# An Implantable Sensorized Lead for Continuous Monitoring of Cardiac Apex Rotation

**DOI:** 10.3390/s18124195

**Published:** 2018-11-30

**Authors:** Emanuela Marcelli, Laura Cercenelli

**Affiliations:** Laboratory of Bioengineering, DIMES Department, University of Bologna, S. Orsola-Malpighi Hospital, 40138 Bologna, Italy; emanuela.marcelli@unibo.it

**Keywords:** cardiac rotation, gyroscopes, implantable lead, heart failure, continuous monitoring

## Abstract

Changes in the pattern or amplitude of cardiac rotation have been associated with important cardiovascular diseases, including Heart Failure (HF) which is one of the major health problems worldwide. Recent advances in echocardiographic techniques have allowed for non-invasive quantification of cardiac rotation; however, these examinations do not address the continuous monitoring of patient status. We have presented a newly developed implantable, transvenous lead with a tri-axis (3D) MEMS gyroscope incorporated near its tip to measure cardiac apex rotation in the three-dimensional space. We have named it CardioMon for its intended use for cardiac monitoring. If compared with currently proposed implantable systems for HF monitoring based on the use of pressure sensors that can have reliability issues, an implantable motion sensor like a gyroscope holds the premise for more reliable long term monitoring. The first prototypal assembly of the CardioMon lead has been tested to assess the reliability of the 3D gyroscope readings. In vitro results showed that the novel sensorized CardioMon lead was accurate and reliable in detecting angular velocities within the range of cardiac twisting velocities. Animal experiments will be planned to further evaluate the CardioMon lead in in vivo environments and to investigate possible endocardial implantation sites.

## 1. Introduction

Cardiac torsion is the wringing motion of the heart around its long axis created by oppositely directed apical and basal rotations and is determined by contracting myofibers, which are arranged in opposite directions between the subendocardial and subepicardial layers [1]. This motion is essential for regulating cardiac systolic and diastolic functions and changes in the pattern or amplitude of cardiac rotation have been associated with various cardiovascular diseases, such as hypertrophic or dilated cardiomyopathy, aortic stenosis, myocardium ischemia, acute myocardial infarction, and Heart Failure (HF) which one of the major health problems worldwide [1,2,3].

In the past cardiac rotation has been quantified using different methods, including multiple implanted markers and biplane cine angiography in transplanted hearts [4,5] and optical devices [6]. More recently, the availability of advanced non-invasive imaging techniques such as tagged magnetic resonance imaging, Doppler tissue imaging, and speckle tracking imaging have expanded the understanding of the relationships between cardiac rotation and cardiac diseases. However, such noninvasive examinations can be used only on an intermittent basis and are not intended to continuously monitor cardiac rotation.

To date, the most significant advancement in the arena of implantable systems for HF monitoring was taken with a novel, wireless, battery-less, pulmonary artery pressure (PAP) monitoring system called the CardioMEMS HF System (Abbott, Sylmar, CA, USA) [7,8,9]. Other PAP measurement systems are being developed, including a small, implanted sensor that has a battery in the capsule and talks through intrabody communication to a Reveal LINQ insertable cardiac monitoring device (developed by Medtronic, Inc., Minneapolis, MN, USA) [10] and a system (developed by Endotronix, Inc., Woodridge, IL, USA) for measuring PAP. This appears to be similar to the CardioMEMS HF System, except for a different external user interface [10].

Currently, devices to monitor left atrial pressure (LAP), which is a direct reflection of left ventricular filling pressure and therefore the primary pressure target for the HF management, are also under development. The HeartPOD (Abbott, formerly St. Jude Medical/Savacor, Inc., Abbott Park, IL, USA), a system that allowed for direct measurement of LAP, was studied in a prospective randomized controlled study that was stopped early due to a perceived excess of implant-related complications [11,12]. The V-LAP system (Vectorious Medical Technologies, Tel Aviv, Israel) is a next generation of implantable LAP monitoring systems, which uses a miniature percutaneous wireless and leadless LAP sensor associated with an advanced, onboard, application-specific, integrated circuit that incorporates a novel drift compensation mechanism [10]. Another micro-electromechanical systems–based LAP monitoring system (Integrated Sensing Systems, Inc., Ypsilanti, MI, USA) has been evaluated in first-in-man studies in patients undergoing implantation of a left ventricular assist device or other cardiac surgery [13]. Some other examples of wireless implants for cardiac monitoring are related to smart stents equipped with microscale sensors [14,15].

All these implantable HF monitoring systems currently under development rely on hemodynamic (pressure) sensors that may have reliability problems in the long-term since the fibrous tissue encapsulation may interfere with the correct operation of the deformable diaphragms or pressure-sensitive surfaces they use.

Monitoring cardiac kinematics rather than hemodynamics holds the premise for early detection of impairment in cardiac function that may predict decompensation in HF patients and prevent recurrent hospitalizations by tailoring an effective drug therapy. Cardiac kinematics can be detected using implantable inertial sensors like gyroscopes and accelerometers. Some researchers have proposed and evaluated the use of miniaturized accelerometers to monitor cardiac function and automatically detect ischemic events by measuring linear displacements in the 3D space [16,17]. However, one of their major concerns is that accelerometers cannot differentiate between acceleration due to motion and acceleration due to gravity, therefore gravity filtering is essential for an accurate motion calculation.

Our research group proposed, for the first time, the use of a gyro sensor, based on Coriolis force, to quantify cardiac apex rotation [18]. Our previous in vivo animal experience was mainly focused on the use of a single-axis (1D) gyro sensor positioned at the epicardial site to detect cardiac rotation during both the normal heart functioning and the experimentally-induced, altered cardiac conditions [19,20,21,22,23].

In this paper, we present the CardioMon, a novel, implantable, sensorized lead equipped with a tri-axis MEMS gyro sensor (3D gyro), intended to continuously monitor cardiac rotation both at epicardial and endocardial sites. Here, we describe the first prototypal assembly of the CardioMon lead and we report the results of preliminary lab tests performed to assess the reliability of the 3D gyro readings.

## 2. Materials and Methods

### 2.1. The Conceptual Design

**Figure 1:**
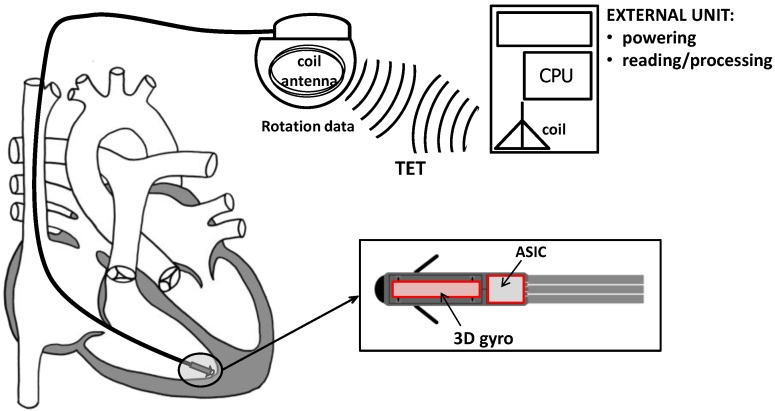
Scheme of conceptual design of the CardioMon lead system for continuous monitoring of cardiac apex rotation.

The conceptual design of the novel CardioMon device for continuous monitoring of cardiac apex rotation is depicted in the scheme of Figure 1. An implantable lead including a 3D gyro and associated ASIC is coupled to a coil antenna, placed in a pacemaker-like case, and positioned subpectorally. The implanted gyro sensor can be powered and interrogated from outside by transcutaneous energy transfer (TET) between the subpectorally implanted coil and the external antenna included in a reading unit held against the patient.

### 2.2. CardioMon Lead Assembly

**Figure 2:**
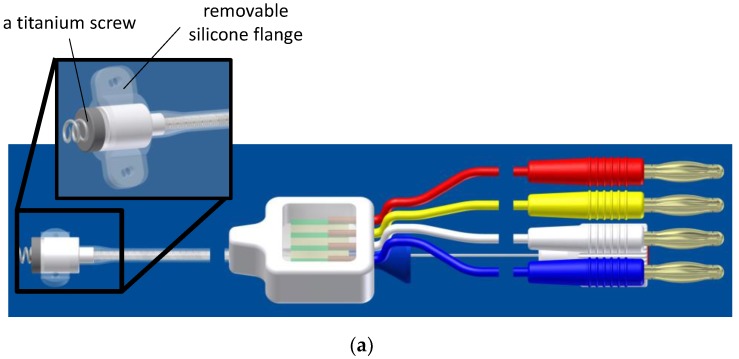

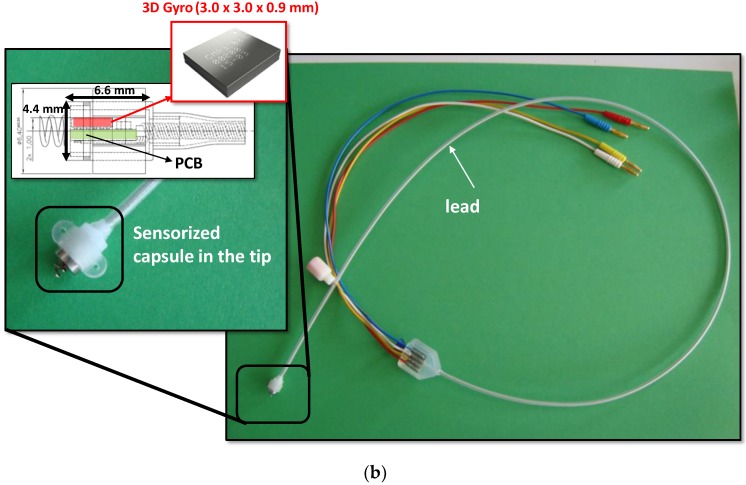
The CardioMon lead: (**a**) 3D rendering of the CardioMon lead design; (**b**) picture of the assembled CardioMon prototype.

The CardioMon lead comprises a sensorized tip and a flexible main body (standard silicone sheath used for implantable cardiac leads), including the electrical connections (4 insulated wires) required for the sensor (Figure 2). The sensorized tip is formed by a Delrin capsule (Ø = 4.4 mm, L = 6.6 mm) that provides a capacitive 3D MEMS gyro sensor (CMR3100-D01, Murata Electronics Oy, Kyoto, Japan) of very small size (3.0 mm *×* 3.0 mm *×* 0.9 mm). It is equipped with a detection range of ±500°/s and a digital SPI/I2C interface for output reading, a miniaturized printed circuit board (PCB), and a titanium screw to allow its secure anchoring to the endocardium. The capsule is also equipped with a removable silicone flange with two holes to allow it to be sutured to the epicardial sites (Figure 2a).

### 2.3. Data Reading Unit

**Figure 3:**
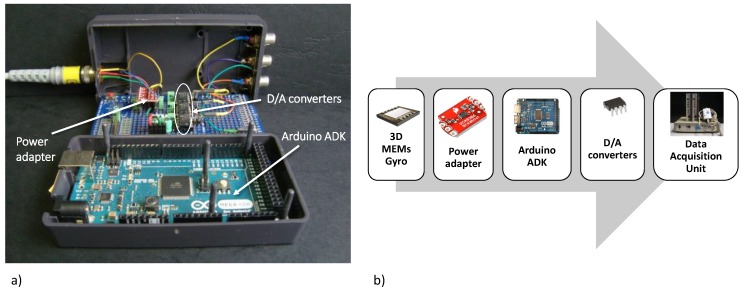
(**a**) Picture and block diagram of (**b**) the data reading unit developed for the output reading of the 3D gyro included in the CardioMon lead.

We assembled a data reading unit to implement the I2C digital communication protocol required for the output reading of the 3D gyro included in the CardioMon lead. The reading unit comprises a microcontroller board (Arduino Mega ADK 5 V/16 MHz, Arduino, Italy), three power adapters, and three Digital/Analog (D/A) converters, one for each sensitive axis of the 3D gyro (Figure 3). The analog output signals from the reading unit have been collected and analyzed using a data acquisition system for biomedical signals (MP100, Biopac System, Goleta, CA, USA).

### 2.4. Functional Lab Tests

Functional lab tests were performed with the assembled CardioMon lead to assess the reliability of the 3D gyro readings when it was encapsulated in the tip of an implantable lead.

**Figure 4:**
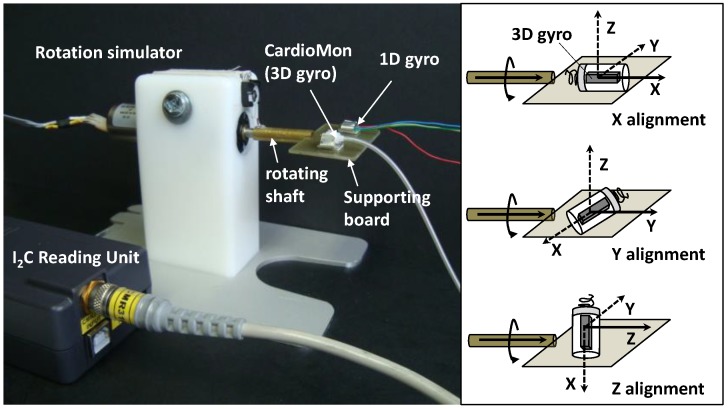
The mechanical simulator with the two mounted gyro sensors.

We developed a mechanical simulator of rotational movements within the range of cardiac rotations to be used for the tests. The simulator was composed of a DC brush motor (Escap 17N78-210E-F16, Portescap, West Chester, PA, USA), controlled by a programmable automation controller (CompactRIO, National Instruments, Austin, TX, USA) and operated by a graphic user interface that we developed in LabVIEW (National Instruments, Austin, TX, USA). A supporting board was anchored to the rotating shaft of the motor and, rotating with it, it was used to position the sensors to be tested (Figure 4).

An “off-the-shelf” single-axis gyro sensor (1D gyro) with analog output (XV-3500CB, Epson Toyocom, Tokyo, Japan) that we had previously evaluated in sheep [21] was used as a reference sensor in the tests.

Both the CardioMon lead including the 3D gyro and the analog 1D gyro were mounted on the supporting board and fixed in stable position by means of adjustable clips attached to the board (Figure 4).

The simulator was programmed to provide target angular velocities (V_T_) in both clockwise (+) and counterclockwise (−) directions (V_T+_ = +200°/s and V_T−_ = −100°/s, respectively) that, according to literature data, can be considered representative of cardiac twisting velocities (i.e., 200°/s in the case of a tachycardia condition and 100°/s in the case of sinus rhythm).

For the tests, the CardioMon lead was fixed on the supporting board of the simulator in order to evaluate the response of each single-axis of the 3D gyro (Figure 4). The detecting axis of the 3D gyro in the tip of the CardioMon lead was aligned with the rotating shaft of the motor (“X alignment”, “Y alignment”, and “Z alignment”, respectively) for each test while the 1D gyro remained in the same position, i.e., with the only one sensitive axis parallel to the shaft.

For each alignment of the CardioMon lead, five repetitions of the following sequence V_0_/V_T+_/V_T−_/V_0_ (with V_0_ = stop motion) were carried out and the angular velocity signals were collected from the two gyro sensors simultaneously.

### 2.5. Data Analysis and Statistics

For each alignment of the CardioMon lead, the three components of the angular velocities detected by each sensitive axis (V_3DX_, V_3DY_, and V_3DZ_) were collected and the modulus of angular velocity (V_3D_), representing the main direction of the angular velocity, was calculated as in the following equation.

(1) $$V_{3D}=\sqrt{V_{3\mathrm{DX}}^{2}+V_{3\mathrm{DY}}^{2}+V_{3\mathrm{DZ}}^{2}}$$

For the 1D gyro, the single-axis velocity (V_1D_) was recorded. V_3DX_, V_3DY_, V_3DZ_, and V_1D_ were considered to be mean ± SD and were obtained by averaging five repetitions of the test sequence with the angular velocity signals detected over the period in which the simulator maintained the set target velocities (V_T+_ and V_T−_).

**Figure 5:**
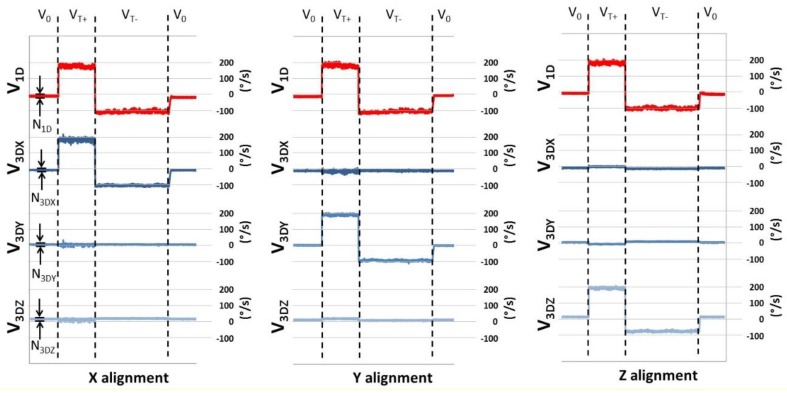
Angular velocity signals recorded with 1D gyro (V_1D_ in red line) and with 3D gyro (three components: V_3DX_, V_3DY_, and V_3DZ_ in blue gradations) during each X, Y, Z alignment; N_1D_ = estimated noise for 1D gyro; N_3DX_, N_3DY_, and N_3DZ_ = estimated noises for each detecting axis, used to obtain the noise level for 3D gyro (N_3D_).

Noise level was estimated for both the 3D gyro (N_3D_) and 1D gyro (N_1D_) as the peak-to-peak angular velocity signal at the V_0_ condition (Figure 5). For the 3D gyro, N_3D_ was identified as the maximum noise level among those detected for each sensitive axis (N_3DX_, N_3DY_, and N_3DZ_). The N_3D_ was estimated for each X, Y, Z alignment.

Measurement errors (E) were calculated for both the 3D gyro (E_3D_) and the 1D gyro (E_1D_) as the difference between the measured angular velocities and the target velocities, by averaging for each X, Y, Z alignment the errors obtained for clockwise and counterclockwise directions.

For comparative evaluations between the 3D gyro (V_3D_) and the 1D gyro (V_1D_) recordings, the Student’s *t*-test was used. All analyses were made with SPSS Version 23.0 (IBM SPSS, New York, NY, USA) and a *p* value of 0.05 was chosen as significant.

## 3. Results

Results from the preliminary in vitro tests showed that the CardioMon lead was well-designed and well-assembled, as well as provided accurate detections of angular velocities within the range of cardiac twisting velocities. After solving initial crosstalk problems related to the arrangement of the 4 wires inside the lead, the angular velocity readings from the 3D gyro included in the sensorized tip were reliable and comparable with detections from the “off-the shelf” 1D gyro. Both were in accordance with the simulated target velocities (Figure 4). For each X, Y, Z alignment of the CardioMon lead, the differences between the mean value of V_3D_ and the corresponding mean value of V_1D_ were not statistically significant for either clockwise or counterclockwise target velocities (Table 1).

**Figure 6:**
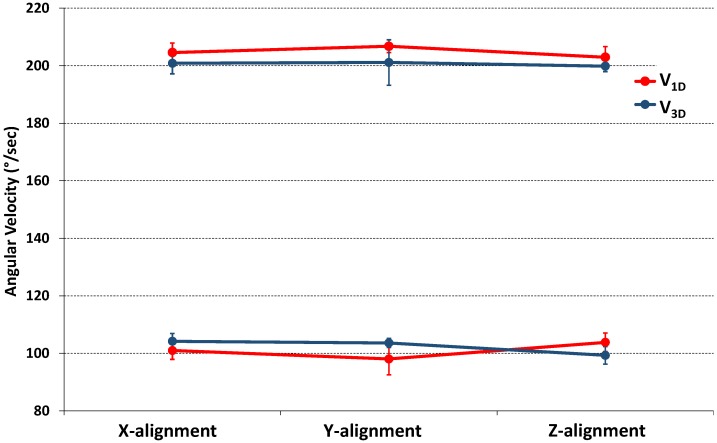
Comparison between the modulus of angular velocities from 3D gyro (V_3D_, in blue line) detected for each X, Y, Z alignment and the single-axis angular velocity from 1D gyro (V_1D_, in red line). Values were reported as mean ± SD from five test repetitions. |V_T+_| = 200°/s; |V_T−_| = 100°/s.

Comparison between the modulus of angular velocities detected by the 3D gyro (V_3D_) for each X, Y, Z alignment and the single-axis angular velocity measured from the 1D gyro are also reported in Figure 6. 

When comparing the 3D gyro recordings with the target velocities programmed for the tests (V_T+_ and V_T−_), we found that, for each X, Y, Z alignment, the measurement errors (E_3D_) were below the corresponding estimated noise level (N_3D_), as well as the measurement error for the 1D gyro (E_1D_) being below the corresponding noise level (N_1D_) (Table 2).

## 4. Discussion

In this study, we present CardioMon, an innovative implantable lead including a miniature 3D MEMS gyro sensor to detect cardiac rotation.

Gyro sensors can provide a direct measurement of cardiac mechanics and may potentially offer more long-term reliability than pressure sensors because they can operate inside a rigid hermetic capsule without being affected by the growth of fibrous tissue that occurs in the harsh environment of human body after implantation. Moreover, with gyro sensors the angular displacement signal is directly obtained by a single integration of the measured angular velocity; it is less affected by bias or noise artifacts than the linear displacement obtained by the double integration of accelerometer signals.

The study results demonstrated that integration of a 3D MEMS gyro into a transvenous lead for cardiac implantable electronic devices (CIEDs) is feasible and reliable, particularly since 3D gyro measurements were in accordance with 1D gyro recordings and target velocities. If compared with a 1D gyro, a 3D gyro has the advantage that in the case of future clinical implantation, it could be placed at an arbitrary orientation rather than requiring the alignment of the only sensitive axis, parallel to the cardiac axis, to be confident of maximum signal detection.

In this study, we reported preliminary in vitro evaluations of the new implantable CardioMon lead, tested using a mechanical simulator of cardiac rotational movements. Additional lab tests will be conducted using an upgraded mechanical simulator that we recently designed, which is capable of reproducing all three components of cardiac motion (longitudinal, radial, and rotational) combined into synchronous movements [24].

Animal experiments will also be planned to further evaluate the CardioMon lead in an in vivo environment and to explore different implantation sites for the sensorized lead within the heart. The in vivo results will be compared to outcomes provided by a computational finite element model of cardiac torsion that we developed previously [25].

## 5. Future Perspective for CardioMon Implantation in Humans

In the current prototype of the CardioMon lead, the dimensions of the sensorized capsule are suitable for experimental evaluations in animals (sheep model) but further miniaturization will be necessary before implantation in humans. Future efforts will be in an attempt to assemble a CardioMon lead with a smaller sensorized capsule on the tip, including a gyro sensor smaller than the current one, e.g., the BMG250 ultra-small three-axis angular rate sensor (Bosch Sensortec GmbH, Kusterdingen, Germany). This is, to our knowledge, the smallest (3 mm × 2.5 mm × 0.83 mm) 3D gyro currently available on the market.

In addressing the future of CardioMon implantation in humans, different options could be explored for exploiting the potential of this novel implantable sensor system. These include the possible CardioMon integration with active implantable therapeutic cardiac devices, such as implantable cardioverter defibrillators (ICDs), or devices for cardiac resynchronization therapy (CRTs), as well as its development as a stand-alone monitoring system.

Following the first hypothesis, the CardioMon system would increase the potential of CIEDs by combining therapy delivery with a continuous monitoring feature, which can also be used to enhance the automatic optimization of the device-delivered therapy itself. The integration of CardioMon in an ICD or a CRT device would not increase power requirements since the implantable gyro sensor can be powered using batteries already used for ICDs or CRT devices.

Stand-alone implantable monitors represent a recent way of approaching the treatment of HF by adding value to the monitoring of symptoms and daily weight alone. An example of a lead-based solution for CardioMon implantable system is the one we previously depicted in Figure 1.

**Figure 7:**
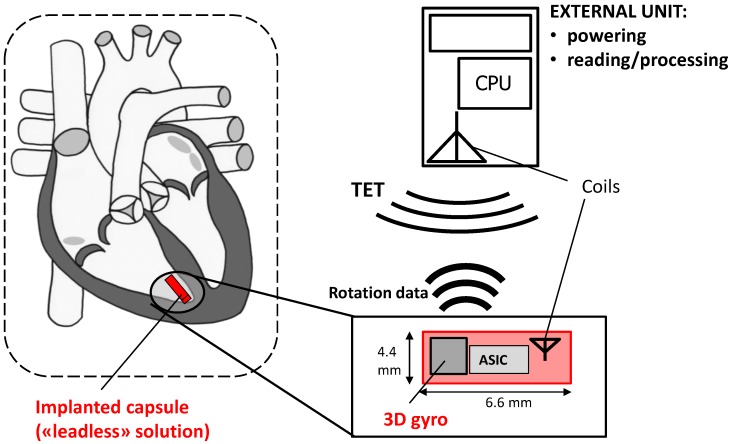
Scheme of a possible *leadless* CardioMon solution designed as a stand-alone implantable cardiac monitor.

A leadless solution could also be pursued for the stand-alone CardioMon system. In this case, following the example of other implantable HF monitoring systems under development [10], the implanted gyro sensor with advanced ASIC would be included in a miniature leadless capsule to be delivered and fixed endocardially at the level of the right ventricular apex (Figure 7). The system would require no leads or batteries and would be concurrently powered and interrogated via an external antenna, similar to the already developed CardioMEMS PAP sensor [7].

## Figures and Tables

**Table 1:** Results of *t*-test analysis used for comparative evaluations between 3D gyro (V_3D_) and 1D gyro (V_1D_) recordings. *p* value < 0.05 indicates statistically significant differences.

	V_T_ [°/s]	V_3D_ [°/s]	V_1D_ [°/s]	*p*
X-alig	|V_T+_| = 200	201 ± 4	205 ± 3	0.218
	|V_T−_| = 100	104 ± 3	101 ± 3	0.065
Y-alig	|V_T+_| = 200	201 ± 8	207 ± 2	0.274
	|V_T−_| = 100	104 ± 2	98 ± 5	0.071
Z-alig	|V_T+_| = 200	199 ± 2	203 ± 4	0.140
	|V_T−_| = 100	99 ± 3	104 ± 3	0.171

**Table 2:** Angular velocity signals, noises, and errors provided by the 3D gyro included in the CardioMon lead and in reference to the 1D gyro.

	Target	3D MEMS Gyro			1D Gyro		
	V_T_ [°/s]	V_3D_ [°/s]	E_3D_ [°/s]	N_3D_ [°/s]	V_1D_ [°/s]	E_1D_ [°/s]	N_1D_ [°/s]
X-alig	V_0_ = 0			7 ± 1			8 ± 1
	|V_T+_| = 200	201 ± 4	4 ± 2		205 ± 3	4 ± 3	
	|V_T−_| = 100	104 ± 3			101 ± 3		
Y-alig	V_0_ = 0			7 ± 1			8 ± 1
	|V_T+_| = 200	201 ± 8	5 ± 4		207 ± 2	6 ± 3	
	|V_T−_| = 100	104 ± 2			98 ± 5		
Z-alig	V_0_ = 0			7 ± 0			10 ± 2
	|V_T+_| = 200	199 ± 2	2 ± 1		203 ± 4	4 ± 3	
	|V_T−_| = 100	99 ± 3			104 ± 3		

X, Y, Z-alig. = alignment of x, y or z-axis of the 3D gyro parallel to the rotating shaft of the simulator; V_0_ = no motion of the simulator; V_T_ = target angular velocities; V_3D_ = modulus of angular velocity measured by the 3D gyro; V_1D_ = single-axis angular velocity for the 1D gyro; E_3D_ = calculated error for the 3D gyro; E_1D_ = calculated error for the 1D gyro; N_3D_ = estimated noise for the 3D gyro; N_1D_ = estimated noise for the 1D gyro. All variables are reported as mean values obtained by averaging the data of five test repetitions.

## References

[1] Beladan C.C., Călin A., Roşca M., Ginghină C., Popescu B.A. (2014). Left ventricular twist dynamics: Principles and applications. Heart Br. Card. Soc..

[2] Fuchs E., Müller M.F., Oswald H., Thöny H., Mohacsi P., Hess O.M. (2004). Cardiac rotation and relaxation in patients with chronic heart failure. Eur. J. Heart Fail..

[3] Benjamin E.J., Virani S.S., Callaway C.W., Chamberlain A.M., Chang A.R., Cheng S., Chiuve S.E., Cushman M., Delling F.N., Deo R. (2018). Heart Disease and Stroke Statistics-2018 Update: A Report from the American Heart Association. Circulation.

[4] Ingels N.B., Daughters G.T., Stinson E.B., Alderman E.L. (1975). Measurement of midwall myocardial dynamics in intact man by radiography of surgically implanted markers. Circulation.

[5] Hansen D.E., Daughters G.T., Alderman E.L., Stinson E.B., Baldwin J.C., Miller D.C. (1987). Effect of acute human cardiac allograft rejection on left ventricular systolic torsion and diastolic recoil measured by intramyocardial markers. Circulation.

[6] Gibbons Kroeker C.A., Ter Keurs H.E., Knudtson M.L., Tyberg J.V., Beyar R. (1993). An optical device to measure the dynamics of apex rotation of the left ventricle. Am. J. Physiol..

[7] Castro P.F., Concepción R., Bourge R.C., Martínez A., Alcaino M., Deck C., Ferrada M., Alfaro M., Perrone S. (2007). A wireless pressure sensor for monitoring pulmonary artery pressure in advanced heart failure: Initial experience. J. Heart Lung Transplant. Off. Publ. Int. Soc. Heart Transplant..

[8] Verdejo H.E., Castro P.F., Concepción R., Ferrada M.A., Alfaro M.A., Alcaíno M.E., Deck C.C., Bourge R.C. (2007). Comparison of a radiofrequency-based wireless pressure sensor to swan-ganz catheter and echocardiography for ambulatory assessment of pulmonary artery pressure in heart failure. J. Am. Coll. Cardiol..

[9] Abraham W.T., Adamson P.B., Bourge R.C., Aaron M.F., Costanzo M.R., Stevenson L.W., Strickland W., Neelagaru S., Raval N., Krueger S. (2011). Wireless pulmonary artery haemodynamic monitoring in chronic heart failure: A randomised controlled trial. Lancet Lond. Engl..

[10] Abraham W.T., Perl L. (2017). Implantable Hemodynamic Monitoring for Heart Failure Patients. J. Am. Coll. Cardiol..

[11] Ritzema J., Melton I.C., Richards A.M., Crozier I.G., Frampton C., Doughty R.N., Whiting J., Kar S., Eigler N., Krum H. (2007). Direct left atrial pressure monitoring in ambulatory heart failure patients: Initial experience with a new permanent implantable device. Circulation.

[12] Troughton R.W., Ritzema J., Eigler N.L., Melton I.C., Krum H., Adamson P.B., Kar S., Shah P.K., Whiting J.S., Heywood J.T. (2011). Direct left atrial pressure monitoring in severe heart failure: Long-term sensor performance. J. Cardiovasc. Transl. Res..

[13] Hubbert L., Baranowski J., Delshad B., Ahn H. (2017). Left Atrial Pressure Monitoring with an Implantable Wireless Pressure Sensor After Implantation of a Left Ventricular Assist Device. ASAIO J. Am. Soc. Artif. Intern. Organs 1992.

[14] Chen X., Assadsangabi B., Hsiang Y., Takahata K. (2018). Enabling Angioplasty-Ready “Smart” Stents to Detect In-Stent Restenosis and Occlusion. Adv. Sci. Weinh. Baden-Wurtt. Ger..

[15] Chow E.Y., Beier B.L., Francino A., Chappell W.J., Irazoqui P.P. (2009). Toward an implantable wireless cardiac monitoring platform integrated with an FDA-approved cardiovascular stent. J. Interv. Cardiol..

[16] Halvorsen P., Remme E., Espinoza A., Hoff L., Skulstad H., Edvardsen T., Fosse E. (2010). Automatic real-time detection of myocardial ischemia by epicardial accelerometer. J. Thorac. Cardiovasc. Surg..

[17] Halvorsen P.S., Espinoza A., Fleischer L.A., Elle O.J., Hoff L., Lundblad R., Skulstad H., Edvardsen T., Ihlen H., Fosse E. (2008). Feasibility of a three-axis epicardial accelerometer in detecting myocardial ischemia in cardiac surgical patients. J. Thorac. Cardiovasc. Surg..

[18] Marcelli E., Plicchi G., Cercenelli L., Bortolami F. (2005). First experimental evaluation of cardiac apex rotation with an epicardial coriolis force sensor. ASAIO J. Am. Soc. Artif. Intern. Organs 1992.

[19] Marcelli E., Cercenelli L., Plicchi G., Auricchio A. (2010). Evaluation of an Innovative Sensor for Cardiac Apex Rotation Monitoring. Int. J. Artif. Organs.

[20] Marcelli E., Cercenelli L., Parlapiano M., Fumero R., Bagnoli P., Costantino M.L., Plicchi G. (2007). Effect of right ventricular pacing on cardiac apex rotation assessed by a gyroscopic sensor. ASAIO J. Am. Soc. Artif. Intern. Organs 1992.

[21] Cercenelli L., Marcelli E. (2015). Cardiac apex rotation assessed by an implantable gyro sensor: Correlation with a lv pressure-derived myocardial performance index in experimentally induced ischemia. J. Mech. Med. Biol..

[22] Marcelli E., Cercenelli L., Musaico M., Bagnoli P., Costantino M.L., Fumero R., Plicchi G. Assessment of cardiac rotation by means of gyroscopic sensors. Proceedings of the Computers in Cardiology.

[23] Marcelli E., Cercenelli L., Parlapiano M.N., Gianfranchi L., Plicchi G. (2011). A Novel Implantable Sensor to Monitor Both Apical Rotation and Cardiac Phases. Int. J. Artif. Organs.

[24] Cutrì E., Bagnoli P., Marcelli E., Biondi F., Cercenelli L., Costantino M.L., Plicchi G., Fumero R. (2010). A mechanical simulator of cardiac wall kinematics. ASAIO J. Am. Soc. Artif. Intern. Organs 1992.

[25] Bagnoli P., Malagutti N., Gastaldi D., Marcelli E., Lui E., Cercenelli L., Costantino M.L., Plicchi G., Fumero R. (2011). Computational finite element model of cardiac torsion. Int. J. Artif. Organs.

